# Cholecalciferol Supplementation and Transplant Outcomes Across Immunosuppressive Regimens in Renal Transplant Recipients Using the United States Food and Drug Administration Adverse Event Reporting System

**DOI:** 10.7759/cureus.105141

**Published:** 2026-03-12

**Authors:** Toru Ogura, Chihiro Shiraishi, Akie Asakura, Aiko Urawa

**Affiliations:** 1 Clinical Research Support Center, Mie University Hospital, Tsu, JPN; 2 Department of Pharmaceutical Sciences for Health Crisis Management, Faculty of Pharmaceutical Sciences, Fukuoka University, Fukuoka, JPN; 3 Department of Clinical Nutrition, Mie University Hospital, Tsu, JPN; 4 Organ Transplantation Center, Mie University Hospital, Tsu, JPN

**Keywords:** adverse drug reaction reporting systems, cholecalciferol, immunosuppressive agents, treatment outcome, vitamin d deficiency

## Abstract

Background

Renal transplant recipients (RTRs) are highly susceptible to vitamin D deficiency because of chronic kidney disease and recommendations for sun avoidance. Cholecalciferol supplementation is frequently prescribed in combination with immunosuppressive therapy; however, comparative safety and efficacy profiles across immunosuppressive regimens remain insufficiently characterized.

Objectives

This study evaluated disproportionality in the reporting of transplant outcome events associated with cholecalciferol supplementation among RTRs receiving different immunosuppressive regimens, using data from the United States Food and Drug Administration Adverse Event Reporting System (FAERS).

Methodology

FAERS data (2004Q1-2025Q4) were analyzed to identify RTRs with and without cholecalciferol exposure. To minimize confounding from concomitant medications, comparisons were restricted to regimen pairs differing only in cholecalciferol inclusion. Reporting odds ratios (RORs) and adjusted RORs (aRORs) were calculated using univariate and multivariate logistic regression to evaluate disproportionate reporting of transplant outcome events (e.g., complications of transplanted kidney, graft dysfunction, graft loss, rejection).

Results

Among 26,002 RTRs, cholecalciferol-added regimens were compared with their cholecalciferol-naive counterparts within each immunosuppressive regimen. In the calcineurin inhibitor (CNI) + steroid (STR) + antimetabolite (ANT) regimen, cholecalciferol addition was associated with significantly lower reporting of transplant outcome events than CNI + STR + ANT without cholecalciferol (ROR: 0.565 (95% confidence interval (CI): 0.382-0.836), *P* = 0.004; aROR: 0.589 (95% CI: 0.392-0.885), *P* = 0.011). Similarly, in the CNI + STR regimen, cholecalciferol addition was associated with significantly lower reporting of transplant outcome events than CNI + STR without cholecalciferol (ROR: 0.297 (95% CI: 0.093-0.950), *P* = 0.041; aROR: 0.306 (95% CI: 0.095-0.981), *P* = 0.046). In contrast, for the CNI + STR + ANT + monoclonal antibody (MAB) regimen, cholecalciferol addition was associated with higher reporting of transplant outcome events than with CNI + STR + ANT + MAB without cholecalciferol (ROR: 1.683 (95% CI: 1.004-2.823), *P* = 0.048; aROR: 1.871 (95% CI: 1.106-3.165), *P* = 0.019).

Conclusions

This FAERS analysis indicates that cholecalciferol supplementation is associated with differential reporting patterns of transplant outcome events across immunosuppressive regimens in RTRs. Lower reporting was observed in base regimens lacking monoclonal antibodies, while higher reporting emerged in monoclonal antibody-containing regimens. These hypothesis-generating findings highlight the need for confirmation through well-designed prospective studies.

## Introduction

Renal transplant recipients (RTRs) face considerable challenges in maintaining adequate vitamin D levels, with deficiency rates reported as high as 80%-85% [1]. Previous studies have shown that 80%-90% of vitamin D in the human body is synthesized in the skin through exposure to ultraviolet B radiation [2]. In RTRs, vitamin D deficiency has been linked to impaired immune function, increased cardiovascular risk, poor graft survival, and a heightened risk of skin cancer [3]. Consequently, supplementation with cholecalciferol has been increasingly investigated as an adjunct to maintenance immunosuppressive therapy.

RTRs generally receive multidrug immunosuppressive regimens composed of five major drug classes: calcineurin inhibitors (CNIs), steroids (STRs), antimetabolites (ANTs), mechanistic target of rapamycin (mTOR) inhibitors, and monoclonal antibodies (MABs). Unlike the other classes used primarily for maintenance therapy, MABs are mainly employed for induction therapy in the early post-transplant period or for acute rejection management and have also been used for prevention and treatment of coronavirus disease 2019 in high-risk patients [4]. CNIs, STRs, ANTs, and mTOR inhibitors are typically used for long-term maintenance. Combining these agents with cholecalciferol introduces potential pharmacological interactions that may modify therapeutic efficacy and safety. Evidence suggests that cholecalciferol may reduce rejection rates, improve allograft function [5], and mitigate post-transplant bone loss [6] when used with certain immunosuppressants. However, the overall impact of cholecalciferol varies across regimens, reflecting possible additive, synergistic, or antagonistic effects [7]. Synergistic effects have been reported between cholecalciferol and STRs for bone metabolism and inflammation [8], and with CNIs for T-cell regulation and metabolic modulation [9]; potential interactions with mTOR inhibitors and MABs have also been theorized, although supporting data remain limited [10]. Because each immunosuppressant class differs in mechanism of action and pharmacokinetic profile, regimen complexity increases further when cholecalciferol is added, potentially altering both efficacy and safety outcomes [11]. Conducting prospective clinical trials for every possible regimen combination is impractical. Therefore, this study employed the United States Food and Drug Administration Adverse Event Reporting System (FAERS) [12] to evaluate safety and efficacy signals associated with cholecalciferol supplementation across immunosuppressive regimens at the drug class level. Although FAERS primarily serves as a pharmacovigilance database, it allows the examination of supplement-related reports and enables paired comparisons of regimens differing only by cholecalciferol inclusion. Despite inherent limitations, such as voluntary reporting bias, incomplete data, and underrepresentation of supplements, the FAERS database provides a large-scale, globally sourced dataset that offers insights unattainable in smaller clinical studies.

The primary objective of this study was to assess the impact of cholecalciferol addition to various immunosuppressive regimens in RTRs using FAERS data, thereby generating evidence to inform clinical decision-making and optimize post-transplant outcomes.

## Materials and methods

Data source

We utilized the FAERS database, a publicly accessible pharmacovigilance database that has been updated quarterly since the first quarter of 2004 (2004Q1). The database was originally known as the Adverse Event Reporting System (AERS) and was rebranded as FAERS in 2012Q4, with expanded and standardized data structures. On January 31, 2026, we downloaded both AERS (aers_ascii_yyyyQq.zip) and FAERS (faers_ascii_yyyyQq.zip) data files covering the period from 2004Q1 through 2025Q4 from the official FAERS website, where "yyyy" and "q" indicate the year and quarter, respectively. To ensure consistency between AERS and FAERS, variable mappings were adjusted according to official documentation. The analysis focused on five of the seven FAERS data files: DEMOyyQq.txt (patient demographic and administrative information), DRUGyyQq.txt (drug information), INDIyyQq.txt (indications for use), REACyyQq.txt (adverse event information), and THERyyQq.txt (drug therapy start and end dates), where "yy" represents the last two digits of the year. In the FAERS database, when a case is updated, a new record is created with an incremented (caseversion) rather than replacing the previous entry. Accordingly, for each patient, only the record with the highest (caseversion) was retained. Because the AERS database does not include the (caseversion) variable, we used the (ISR) and (CASE) variables for analogous case version identification. Throughout this manuscript, FAERS variable names are denoted in curly braces. Before statistical analyses, data cleaning was performed to standardize measurement units and correct irregular entries for four essential background variables: (sex), patient age at event (age), (weight), and reporter's country (reporter_country) (Appendix A). Additionally, missing line breaks identified in specific AERS files were manually corrected to prevent parsing errors. Specifically, line breaks were restored at lines 322,967 in DRUG11Q2.txt, 247,896 in DRUG11Q3.txt, and 446,738 in DRUG11Q4.txt.

As FAERS is a publicly available, de-identified, and unlinkable database, institutional review board approval was not required for this study.

Study design

The inclusion criteria were individual case safety reports submitted to FAERS between 2004Q1 and 2025Q4 in which one or more immunosuppressive agents were administered for renal transplantation. Immunosuppressive exposure was defined at the drug‑class level and included the following classes: CNI (tacrolimus and cyclosporine), STR (prednisone, prednisolone, and methylprednisolone), ANT (mycophenolic acid, mycophenolate mofetil, mycophenolate sodium, azathioprine, and mizoribine), mTOR inhibitors (everolimus), and MAB (rituximab and basiliximab). These agents were identified by their generic names in the (prod_ai) field in FAERS. For AERS, which does not contain (prod_ai), both generic and brand names were extracted from the (drugname) field (Appendix B). Cases were determined using the indication field: reports were considered RTRs when the indication (indi_pt) explicitly contained "renal transplantation" as the indication for the reported drug therapy.

The exclusion criteria were as follows. First, cases in which immunosuppressive therapy was initiated only after the occurrence of adverse events (AEs), as judged from the therapy start and event dates, were excluded. Second, because FAERS accepts reports from multiple types of reporters, potential duplicate reports for the same individual were identified by matching patient characteristics, treatment regimens, and AE information; when duplicates were suspected, only one record was retained per patient (Appendix C). Finally, treatment regimen pairs were included in the disproportionality analysis only when both the cholecalciferol‑added and cholecalciferol‑naive groups contained sufficient sample sizes; regimen pairs with <40 cases in either group were excluded. All reports that met the inclusion criteria and did not meet any exclusion criteria were included in the analysis; therefore, no additional sampling or active recruitment of participants was undertaken, reflecting the database‑based nature of the study.

Base regimens were defined as specific combinations of these classes. Each regimen was stratified according to cholecalciferol exposure into cholecalciferol-added (with cholecalciferol) and cholecalciferol-naive (without cholecalciferol) groups. This stratification enabled direct comparison of outcomes associated with cholecalciferol supplementation while minimizing potential confounding arising from differences in underlying regimens.

Efficacy and safety endpoints were based on AEs coded using the Medical Dictionary for Regulatory Activities preferred terms (pt). These AEs were grouped into clinically relevant categories, including transplant outcome events, infections, cancer, renal disorders, musculoskeletal and mobility disorders, general disorders, gastrointestinal disorders, cardiovascular disorders, hematologic disorders, nervous system disorders, and respiratory disorders (specific terms are listed in Appendix D). Because immunosuppressive therapy primarily aims to prevent transplant outcome events, the frequency of these reports was used as a surrogate measure of efficacy. Although FAERS is principally designed for pharmacovigilance, its capture of therapeutic failures allowed secondary evaluation of efficacy. This dual analytical approach provided a comprehensive assessment of both the efficacy and safety of each regimen.

Statistical analyses

Continuous variables were summarized as medians with first and third quartiles, and categorical variables were summarized as frequencies with reporting proportions (RPs) [13]. RP was calculated as (number of RTRs with the characteristic of interest)/(total RTRs reported to FAERS) × 100. Reporting odds ratios (RORs) [14] and corresponding 95% confidence intervals (CIs) were estimated using univariate binomial logistic regression. Adjusted RORs (aRORs) and corresponding 95% CIs were calculated using multivariate binomial logistic regression to account for differences in patient characteristics. Because of the high RP of unknown values for (weight), this variable was excluded from both ROR and aROR analyses. Covariates ((age), (sex), and (reporter_country)) were selected through a stepwise variable selection procedure. Missing data were not imputed because FAERS reports often contain extensive and non-random missingness in key patient characteristics, which could have introduced bias if imputation had been applied. Because the number of candidate covariates was small and missingness was substantial, we fitted all possible subsets of covariates. For each candidate model, logistic regression was performed using all observations with non‑missing data on the covariates included in that model. The final model was chosen by jointly considering the effective sample size and the statistical significance of the covariates. For each immunosuppressive regimen, outcomes in cholecalciferol-added groups (with cholecalciferol) were compared with those in cholecalciferol-naive reference groups (without cholecalciferol). This matched-pairs comparison approach minimized regimen-specific confounding and isolated potential associations attributable to cholecalciferol supplementation. The (reporter_country) variable was categorized into three regions-North America, Europe, and other continents-according to major reporting patterns. Reference categories were set as female for (sex) and other continents for (reporter_country). A two-sided *P*-value < 0.05 was considered statistically significant. Given that many cases were excluded from multivariate analyses due to missing data, we considered findings to be robust only when both the ROR and aROR were statistically significant. All statistical analyses were conducted using R software version 4.4.1 (R Foundation for Statistical Computing, Vienna, Austria).

## Results

Patient background

**Figure 1:**
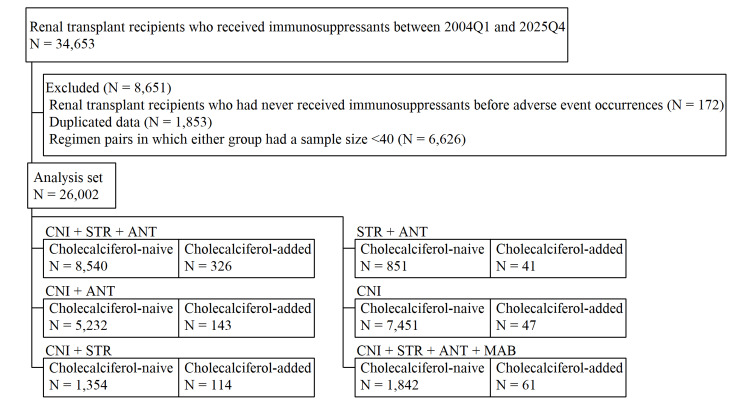
Flowchart of renal transplant recipients who received immunosuppressants. ANT, antimetabolite; CNI, calcineurin inhibitors; MAB, monoclonal antibody; STR, steroid

A total of 34,653 RTRs receiving immunosuppressive therapy between 2004Q1 and 2025Q4 were initially identified in the FAERS database. After applying the predefined exclusion criteria, 26,002 RTRs remained in the final analysis set, and 15,852 cases were excluded. Figure 1 presents the distribution of immunosuppressive regimen combinations within the dataset and displays paired regimen groups side by side, differing only in the inclusion or exclusion of cholecalciferol.

Table 1 summarizes the demographic characteristics of RTRs across the various regimen groups. The study population showed diverse demographic profiles, with male patients comprising the majority in most groups. In general, patients receiving cholecalciferol were older than those in the cholecalciferol-naive groups. Geographic variation was also evident: reports for cholecalciferol-naive patients originated from a broader range of countries, whereas reports involving cholecalciferol-added patients were less frequent from South America and Asia.

**Table 1:** Summary of renal transplant recipient characteristics across regimen groups. Sex and country are summarized as frequencies (reporting proportions). Age and weight are summarized as medians and first and third quartiles. Unknown for each variable is summarized as frequency (reporting proportion). ANT, antimetabolites; CNI, calcineurin inhibitors; MAB, monoclonal antibodies; Q1, first quartile; Q3, third quartile; STR, steroids

Base regimen	CNI + STR + ANT		CNI + ANT		CNI + STR		STR + ANT		CNI		CNI + STR + ANT + MAB	
Cholecalciferol	Without	With	Without	With	Without	With	Without	With	Without	With	Without	With
	*N* = 8,540	*N* = 326	*N* = 5,232	*N* = 143	*N* = 1,354	*N* = 114	*N* = 851	*N* = 41	*N* = 7,451	*N* = 47	*N* = 1,842	*N* = 61
Sex, *n* (%)												
Female	3,170 (37.1)	160 (49.1)	2,063 (39.4)	74 (51.7)	542 (40.0)	44 (38.6)	313 (36.8)	23 (56.1)	2,898 (38.9)	16 (34.0)	665 (36.1)	10 (16.4)
Male	4,766 (55.8)	153 (46.9)	2,822 (53.9)	66 (46.2)	705 (52.1)	65 (57.0)	452 (53.1)	17 (41.5)	4,093 (54.9)	30 (63.8)	1,026 (55.7)	48 (78.7)
Unknown	604 (7.1)	13 (4.0)	347 (6.6)	3 (2.1)	107 (7.9)	5 (4.4)	86 (10.1)	1 (2.4)	460 (6.2)	1 (2.1)	151 (8.2)	3 (4.9)
Age (years)												
Median	52.0	57.0	54.0	63.0	56.0	69.0	55.0	58.0	55.0	62.0	48.0	50.0
Q1-Q3	38.0-62.0	41.0-67.0	40.0-65.0	51.8-72.0	41.0-66.0	59.0-76.0	44.0-65.0	54.0-65.0	41.0-64.0	46.0-68.0	33.0-58.0	34.5-61.0
Unknown, *n* (%)	1,262 (14.8)	56 (17.2)	1,374 (26.3)	35 (24.5)	244 (18.0)	21 (18.4)	161 (18.9)	4 (9.8)	2,024 (27.2)	10 (21.3)	229 (12.4)	6 (9.8)
Weight (kg)												
Median	65.0	70.0	69.0	69.5	64.5	69.0	75.0	75.2	64.0	82.0	63.8	88.0
Q1-Q3	55.0-81.7	58.5-84.0	57.0-83.0	60.0-81.8	55.0-77.4	61.0-69.0	61.2-87.0	64.1-83.8	54.0-78.0	65.5-100.0	54.0-75.4	69.0-103.5
Unknown, *n* (%)	6,833 (80.0)	191 (58.6)	4,348 (83.1)	93 (65.0)	1,013 (74.8)	53 (46.5)	615 (72.3)	17 (41.5)	6,162 (82.7)	34 (72.3)	1,461 (79.3)	31 (50.8)
Continent, *n* (%)												
North America	2,808 (32.9)	167 (51.2)	2,323 (44.4)	101 (70.6)	489 (36.1)	45 (39.5)	347 (40.8)	25 (61.0)	3,974 (53.3)	33 (70.2)	299 (16.2)	21 (34.4)
Latin America	537 (6.3)	6 (1.8)	220 (4.2)	1 (0.7)	79 (5.8)	0 (0.0)	65 (7.6)	0 (0.0)	639 (8.6)	0 (0.0)	22 (1.2)	1 (1.6)
Asia	1,549 (18.1)	10 (3.1)	842 (16.1)	2 (1.4)	155 (11.4)	0 (0.0)	106 (12.5)	1 (2.4)	979 (13.1)	2 (4.3)	677 (36.8)	20 (32.8)
Europe	3,233 (37.9)	121 (37.1)	1,568 (30.0)	38 (26.6)	537 (39.7)	66 (57.9)	268 (31.5)	15 (36.6)	1,302 (17.5)	12 (25.5)	722 (39.2)	18 (29.5)
Oceania	47 (0.6)	1 (0.3)	48 (0.9)	0 (0.0)	7 (0.5)	0 (0.0)	2 (0.2)	0 (0.0)	23 (0.3)	0 (0.0)	6 (0.3)	0 (0.0)
Africa	110 (1.3)	7 (2.1)	23 (0.4)	0 (0.0)	20 (1.5)	2 (1.8)	13 (1.5)	0 (0.0)	60 (0.8)	0 (0.0)	52 (2.8)	0 (0.0)
Unknown	256 (3.0)	14 (4.3)	208 (4.0)	1 (0.7)	67 (4.9)	1 (0.9)	50 (5.9)	0 (0.0)	474 (6.4)	0 (0.0)	64 (3.5)	1 (1.6)

Adverse events

Table 2 summarizes AE categories across regimen pairs. Cholecalciferol-added groups generally showed lower reporting proportions for transplant outcome events, infections, and cancer than their cholecalciferol‑naive counterparts across all regimens, except for transplant outcome events in the CNI + STR + ANT + MAB regimen, infections in the CNI + STR regimen, and cancer in the CNI regimen.

**Table 2:** Summary of adverse event categories. Data are summarized as frequencies (reporting proportions). If multiple adverse events are reported in a patient, each adverse event is counted. Each patient is counted only once per adverse event category, regardless of the number of preferred terms experienced within that category. ANT, antimetabolites; CNI, calcineurin inhibitors; MAB, monoclonal antibodies; Q1, first quartile; Q3, third quartile; STR, steroids

Base regimen	CNI + STR + ANT		CNI + ANT		CNI + STR		STR + ANT		CNI		CNI + STR + ANT + MAB	
Cholecalciferol	Without	With	Without	With	Without	With	Without	With	Without	With	Without	With
	*N* = 8,540	*N* = 326	*N* = 5,232	*N* = 143	*N* = 1,354	*N* = 114	*N* = 851	*N* = 41	*N* = 7,451	*N* = 47	*N* = 1,842	*N* = 61
Transplant outcome events	1,218 (14.3)	28 (8.6)	519 (9.9)	5 (3.5)	113 (8.3)	3 (2.6)	89 (10.5)	2 (4.9)	620 (8.3)	1 (2.1)	564 (30.6)	26 (42.6)
Infections	2,441 (28.6)	73 (22.4)	1,040 (19.9)	18 (12.6)	308 (22.7)	30 (26.3)	208 (24.4)	10 (24.4)	878 (11.8)	5 (10.6)	681 (37.0)	11 (18.0)
Cancer	347 (4.1)	7 (2.1)	103 (2.0)	1 (0.7)	40 (3.0)	3 (2.6)	46 (5.4)	1 (2.4)	76 (1.0)	2 (4.3)	76 (4.1)	1 (1.6)
Renal disorders	1,889 (22.1)	85 (26.1)	943 (18.0)	20 (14.0)	256 (18.9)	35 (30.7)	112 (13.2)	3 (7.3)	1,138 (15.3)	10 (21.3)	673 (36.5)	35 (57.4)
Musculoskeletal and mobility disorders	375 (4.4)	16 (4.9)	228 (4.4)	15 (10.5)	71 (5.2)	4 (3.5)	40 (4.7)	1 (2.4)	294 (3.9)	3 (6.4)	61 (3.3)	2 (3.3)
General disorders	1,128 (13.2)	47 (14.4)	607 (11.6)	22 (15.4)	163 (12.0)	35 (30.7)	100 (11.8)	8 (19.5)	693 (9.3)	3 (6.4)	258 (14.0)	11 (18.0)
Gastrointestinal disorders	1,158 (13.6)	50 (15.3)	693 (13.2)	18 (12.6)	166 (12.3)	29 (25.4)	108 (12.7)	7 (17.1)	749 (10.1)	3 (6.4)	256 (13.9)	18 (29.5)
Cardiovascular disorders	700 (8.2)	47 (14.4)	507 (9.7)	18 (12.6)	168 (12.4)	27 (23.7)	59 (6.9)	4 (9.8)	672 (9.0)	7 (14.9)	154 (8.4)	5 (8.2)
Hematologic disorders	885 (10.4)	54 (16.6)	420 (8.0)	8 (5.6)	118 (8.7)	5 (4.4)	86 (10.1)	3 (7.3)	324 (4.3)	2 (4.3)	371 (20.1)	16 (26.2)
Nervous system disorders	459 (5.4)	24 (7.4)	254 (4.9)	6 (4.2)	84 (6.2)	6 (5.3)	25 (2.9)	4 (9.8)	436 (5.9)	2 (4.3)	73 (4.0)	6 (9.8)
Respiratory disorders	876 (10.3)	33 (10.1)	404 (7.7)	12 (8.4)	145 (10.7)	27 (23.7)	92 (10.8)	2 (4.9)	429 (5.8)	7 (14.9)	189 (10.3)	7 (11.5)

Table 3 presents RORs and aRORs for transplant outcome events. The cholecalciferol-added CNI + STR + ANT regimen showed significantly lower disproportionality than CNI + STR + ANT without cholecalciferol (ROR: 0.565 (95% CI 0.382-0.836), *P* = 0.004; aROR: 0.589 (95% CI 0.392-0.885), *P* = 0.011). The cholecalciferol-added CNI + STR regimen also showed significantly lower disproportionality than CNI + STR (ROR: 0.297 (95% CI 0.093-0.950), *P* = 0.041; aROR: 0.306 (95% CI 0.095-0.981), *P* = 0.046). Cholecalciferol-added CNI + ANT was associated with lower disproportionality than CNI + ANT (ROR: 0.329 (95% CI 0.134-0.806), *P* = 0.015), although this association was not significant after adjustment (aROR: 0.442 (95% CI 0.179-1.090), *P* = 0.076). In contrast, the cholecalciferol-added CNI + STR + ANT + MAB regimen showed significantly higher disproportionality than CNI + STR + ANT + MAB (ROR: 1.683 (95% CI 1.004-2.823), *P* = 0.048; aROR: 1.871 (95% CI 1.106-3.165), *P* = 0.019).

**Table 3:** ROR and aROR for transplant outcome events. RORs were estimated using univariate binomial logistic regression. aRORs were estimated using multivariate binomial logistic regression with covariates selected by a stepwise variable selection procedure. -, not selected in aROR (variable selection was performed). ANT, antimetabolites; aROR, adjusted reporting odds ratio; CI, confidence interval; CNI, calcineurin inhibitors; MAB, monoclonal antibodies; Q1, first quartile; Q3, third quartile; ROR, reporting odds ratio; STR, steroids

Variable	Reference	Comparison	ROR (95% CI)	p-value	aROR (95% CI)	p-value
Regimen	CNI + STR + ANT	Cholecalciferol-added	0.565 (0.382-0.836)	0.004	0.589 (0.392-0.885)	0.011
Sex	Female	Male	1.158 (1.017-1.318)	0.027	-	-
Age	Per one-year increase		0.978 (0.974-0.981)	<0.001	-	-
Continent	Other continents	North America	0.709 (0.609-0.825)	<0.001	0.723 (0.620-0.843)	<0.001
Continent	Other continents	Europe	0.682 (0.588-0.792)	<0.001	0.690 (0.594-0.801)	<0.001
Regimen	CNI + ANT	Cholecalciferol-added	0.329 (0.134-0.806)	0.015	0.442 (0.179-1.090)	0.076
Sex	Female	Male	0.984 (0.810-1.195)	0.870	-	-
Age	Per one-year increase		0.977 (0.971-0.982)	<0.001	-	-
Continent	Other continents	North America	0.312 (0.246-0.398)	<0.001	0.320 (0.251-0.407)	<0.001
Continent	Other continents	Europe	0.802 (0.644-0.999)	0.049	0.812 (0.652-1.012)	0.063
Regimen	CNI + STR	Cholecalciferol-added	0.297 (0.093-0.950)	0.041	0.306 (0.095-0.981)	0.046
Sex	Female	Male	1.624 (1.068-2.469)	0.023	1.638 (1.077-2.492)	0.021
Age	Per one-year increase		0.986 (0.975-0.997)	0.012	-	-
Continent	Other continents	North America	0.497 (0.293-0.844)	0.010	-	-
Continent	Other continents	Europe	0.714 (0.440-1.158)	0.172	-	-
Regimen	STR + ANT	Cholecalciferol-added	0.439 (0.104-1.849)	0.262	0.750 (0.174-3.226)	0.699
Sex	Female	Male	1.142 (0.684-1.907)	0.611	-	-
Age	Per one-year increase		0.977 (0.962-0.993)	0.004	0.977 (0.962-0.993)	0.004
Continent	Other continents	North America	1.091 (0.622-1.915)	0.761	-	-
Continent	Other continents	Europe	0.704 (0.373-1.329)	0.279	-	-
Regimen	CNI	Cholecalciferol-added	0.240 (0.033-1.740)	0.158	0.321 (0.043-2.378)	0.266
Sex	Female	Male	0.985 (0.829-1.171)	0.867	-	-
Age	Per one-year increase		0.969 (0.964-0.974)	<0.001	0.969 (0.964-0.974)	<0.001
Continent	Other continents	North America	0.547 (0.448-0.668)	<0.001	-	-
Continent	Other continents	Europe	0.899 (0.709-1.140)	0.378	-	-
Regimen	CNI + STR + ANT + MAB	Cholecalciferol-added	1.683 (1.004-2.823)	0.048	1.871 (1.106-3.165)	0.019
Sex	Female	Male	1.046 (0.849-1.288)	0.675	-	-
Age	Per one-year increase		0.994 (0.988-1.000)	0.052	-	-
Continent	Other continents	North America	0.641 (0.479-0.859)	0.003	0.623 (0.464-0.836)	0.002
Continent	Other continents	Europe	0.775 (0.624-0.963)	0.021	0.776 (0.624-0.964)	0.022

Table 4 presents RORs and aRORs for infections. The cholecalciferol-added CNI + STR + ANT regimen had significantly lower disproportionality than CNI + STR + ANT (ROR: 0.721 (95% CI 0.553-0.939), *P* = 0.015; aROR: 0.737 (95% CI 0.560-0.970), *P* = 0.029), and the cholecalciferol-added CNI + STR + ANT + MAB regimen also had significantly lower disproportionality than CNI + STR + ANT + MAB (ROR: 0.375 (95% CI 0.194-0.725), *P* = 0.004; aROR 0.382 (95% CI 0.197-0.741), *P* = 0.004).

**Table 4:** ROR and aROR for infections. RORs were estimated using univariate binomial logistic regression. aRORs were estimated using multivariate binomial logistic regression with covariates selected by a stepwise variable selection procedure. -, not selected in aROR (variable selection was performed). ANT, antimetabolites; aROR, adjusted reporting odds ratio; CI, confidence interval; CNI, calcineurin inhibitors; MAB, monoclonal antibodies; Q1, first quartile; Q3, third quartile; ROR, reporting odds ratio; STR, steroids

Variable	Reference	Comparison	ROR (95% CI)	*P*-value	aROR (95% CI)	*P*-value
Regimen	CNI + STR + ANT	Cholecalciferol-added	0.721 (0.553-0.939)	0.015	0.737 (0.560-0.970)	0.029
Sex	Female	Male	0.967 (0.878-1.066)	0.502	-	-
Age	Per one-year increase		1.002 (1.000-1.005)	0.103	-	-
Continent	Other continents	North America	0.732 (0.649-0.826)	<0.001	0.741 (0.657-0.837)	<0.001
Continent	Other continents	Europe	0.831 (0.740-0.933)	0.002	0.837 (0.745-0.939)	0.003
Regimen	CNI + ANT	Cholecalciferol-added	0.580 (0.352-0.956)	0.033	0.626 (0.379-1.032)	0.066
Sex	Female	Male	0.975 (0.848-1.122)	0.728	-	-
Age	Per one-year increase		1.000 (0.995-1.004)	0.941	-	-
Continent	Other continents	North America	0.696 (0.586-0.826)	<0.001	0.707 (0.595-0.840)	<0.001
Continent	Other continents	Europe	0.817 (0.680-0.981)	0.030	0.824 (0.686-0.989)	0.038
Regimen	CNI + STR	Cholecalciferol-added	1.213 (0.784-1.875)	0.385	1.219 (0.781-1.904)	0.384
Sex	Female	Male	1.345 (1.038-1.743)	0.025	1.343 (1.036-1.740)	0.026
Age	Per one-year increase		1.001 (0.994-1.009)	0.750	-	-
Continent	Other continents	North America	0.822 (0.577-1.172)	0.278	-	-
Continent	Other continents	Europe	1.055 (0.751-1.481)	0.759	-	-
Regimen	STR + ANT	Cholecalciferol-added	0.997 (0.481-2.069)	0.994	0.997 (0.481-2.069)	0.994
Sex	Female	Male	1.011 (0.729-1.402)	0.948	-	-
Age	Per one-year increase		0.999 (0.989-1.009)	0.824	-	-
Continent	Other continents	North America	1.078 (0.725-1.604)	0.710	-	-
Continent	Other continents	Europe	0.619 (0.397-0.966)	0.035	-	-
Regimen	CNI	Cholecalciferol-added	0.891 (0.352-2.259)	0.808	1.055 (0.415-2.685)	0.910
Sex	Female	Male	0.858 (0.742-0.991)	0.038	0.815 (0.700-0.949)	0.008
Age	Per one-year increase		0.998 (0.993-1.002)	0.350	-	-
Continent	Other continents	North America	0.568 (0.481-0.672)	<0.001	0.554 (0.467-0.658)	<0.001
Continent	Other continents	Europe	0.687 (0.555-0.851)	0.001	0.705 (0.565-0.880)	0.002
Regimen	CNI + STR + ANT + MAB	Cholecalciferol-added	0.375 (0.194-0.725)	0.004	0.382 (0.197-0.741)	0.004
Sex	Female	Male	1.023 (0.838-1.250)	0.820	-	-
Age	Per one-year increase		1.004 (0.999-1.010)	0.136	-	-
Continent	Other continents	North America	1.155 (0.880-1.516)	0.299	1.193 (0.907-1.568)	0.207
Continent	Other continents	Europe	1.299 (1.053-1.602)	0.015	1.297 (1.051-1.601)	0.015

Table 5 summarizes safety signals where both ROR >1 and aROR >1 for various AE categories: renal and gastrointestinal disorders (CNI + STR + ANT + MAB), general disorders (CNI + STR), hematologic disorders (CNI + STR + ANT), nervous system disorders (STR + ANT), and cancer (CNI).

**Table 5:** Disproportionality signals for adverse event categories by immunosuppressive regimen. ROR and aROR comparing cholecalciferol-added vs. cholecalciferol-naive (reference) groups within each base regimen were estimated using univariate and multivariable binomial logistic regression, respectively. >1: Both ROR and aROR significantly >1; N.S.: At least one of ROR and aROR was not statistically significant (including cases in which only one or neither was significant). ANT, antimetabolites; aROR, adjusted reporting odds ratio; CNI, calcineurin inhibitors; MAB, monoclonal antibodies; ROR, reporting odds ratio; STR, steroids

Adverse event category	CNI + STR + ANT	CNI + ANT	CNI + STR	STR + ANT	CNI	CNI + STR + ANT + MAB
Cancer	N.S.	N.S.	N.S.	N.S.	>1	N.S.
Renal disorders	N.S.	N.S.	>1	N.S.	N.S.	>1
Musculoskeletal and mobility disorders	N.S.	>1	N.S.	N.S.	N.S.	N.S.
General disorders	N.S.	N.S.	>1	N.S.	N.S.	N.S.
Gastrointestinal disorders	N.S.	N.S.	>1	N.S.	N.S.	>1
Cardiovascular disorders	>1	N.S.	>1	N.S.	N.S.	N.S.
Hematologic disorders	>1	N.S.	N.S.	N.S.	N.S.	N.S.
Nervous system disorders	N.S.	N.S.	N.S.	>1	N.S.	>1
Respiratory disorders	N.S.	N.S.	>1	N.S.	N.S.	N.S.

## Discussion

This FAERS analysis represents a large-scale pharmacovigilance investigation examining cholecalciferol supplementation across diverse immunosuppressive regimens in RTRs. The key finding is a differential pattern of transplant outcome event reporting: cholecalciferol addition was associated with significantly lower reporting in the CNI + STR + ANT regimen - the most common maintenance therapy - while higher reporting emerged specifically in regimens containing MABs. These findings align with vitamin D's established immunomodulatory properties while highlighting regimen-specific safety considerations [15].

The observed reduction in transplant outcome events with cholecalciferol addition to CNI + STR + ANT corroborates preclinical and smaller clinical studies suggesting beneficial effects on graft function [5]. Vitamin D modulates T-cell differentiation and regulatory T-cell function [16], potentially synergizing with calcineurin inhibitors to suppress alloimmune responses. Prior observational data also link higher 25-hydroxyvitamin D levels with improved estimated glomerular filtration rates at 12 months post-transplant [3]. Although our analysis cannot establish causality, the consistent (albeit nonsignificant) trend toward lower reporting across most base regimens supports a potential protective signal warranting prospective validation. Conversely, the increased reporting of transplant outcome events in MAB-containing regimens raises important safety concerns. Monoclonal antibodies, primarily used for induction or rejection treatment, identify higher-risk RTRs with complex immunological profiles. Cholecalciferol may interact differently in this context, potentially through heightened calcium-mediated effects or altered pharmacokinetics in more severely ill patients. Notably, safety signals were also identified for renal and gastrointestinal disorders (CNI + STR + ANT + MAB), hematologic disorders (CNI + STR + ANT), and other categories, emphasizing the need for vigilant monitoring when combining cholecalciferol with multidrug regimens. Several secondary findings merit consideration. Cholecalciferol-added groups demonstrated lower infection reporting across most regimens, consistent with vitamin D's antimicrobial peptide induction and immune regulatory effects [7]. Similarly, reduced cancer reporting aligns with epidemiological data linking adequate vitamin D status to lower malignancy risk in RTRs [17]. However, increased reporting of specific AEs (e.g., renal, hematologic disorders) underscores known risks of vitamin D excess, including hypercalcemia and tissue calcification, particularly when combined with steroids and calcineurin inhibitors. These pharmacovigilance signals complement, but extend beyond, randomized controlled trial (RCT) evidence. Existing RCTs have primarily examined cholecalciferol in standard triple therapy (CNI + STR + ANT), demonstrating safety and trends toward improved bone health without consistent graft function benefits [5,6]. Our analysis uniquely addresses heterogeneity across regimens, revealing potential risks in MAB-containing protocols - a gap unexamined in prospective trials due to practical constraints.

The demographic composition of our study population aligns well with global RTR patterns. Males predominated across most regimen groups [18], consistent with worldwide transplant demographics. Cholecalciferol-added groups were generally older than cholecalciferol-naive groups, reflecting the higher prevalence of vitamin D deficiency among older RTRs [19]. Geographic reporting patterns revealed less frequent cholecalciferol use in South America and Asia compared with North America and Europe [20,21], mirroring regional differences in vitamin D testing and supplementation practices rather than true prevalence disparities.

Strengths of this study include the regimen-stratified analysis of cholecalciferol supplementation across diverse immunosuppressive protocols using >20 years of global FAERS data (*N* = 26,002 RTRs). The paired comparison design effectively minimized regimen-related confounding, and the class-level stratification provided novel insights unattainable through smaller RCTs.

This study has several limitations inherent to FAERS analyses and to pharmacovigilance study designs in general. First, spontaneous reporting introduces underreporting, reporting heterogeneity, and channeling bias; patients who receive cholecalciferol may differ systematically from cholecalciferol‑naive patients in disease severity, comorbidity burden, access to care, and monitoring practices. These differences may influence both the likelihood of receiving supplementation and the probability of having AEs reported, thereby affecting internal validity. Second, because FAERS lacks reliable denominators and person‑time information, our results reflect disproportionality rather than incidence or risk, and should not be interpreted as absolute rates. Third, important clinical variables, such as graft vintage, HLA mismatch, donor type, baseline kidney function, concomitant immunosuppressive drugs within classes, and cholecalciferol dose and duration, are not systematically captured, resulting in incomplete control of confounding and substantial residual confounding despite multivariable adjustment. Fourth, missing data and incomplete reporting of supplements may attenuate or distort signals, and the available‑case approach used in our multivariable analyses further reduced the effective sample size, particularly in some regimen pairs. Fifth, temporal relationships between cholecalciferol exposure and events cannot always be precisely established, and misclassification of exposure or outcome categories based on coded terms cannot be entirely excluded. Finally, our findings are based on reports submitted predominantly from North America and Europe, with fewer reports from South America and Asia, which may limit generalizability to regions with different transplant practices, vitamin D status, and supplementation policies. Collectively, these limitations indicate that the observed disproportionality signals should be interpreted as hypothesis‑generating and not as definitive evidence of causal effects. To mitigate some of these limitations, we restricted comparisons to regimen pairs differing only in cholecalciferol inclusion, provided detailed operational definitions of exposures, covariates, and outcomes, and considered signals to be robust only when both unadjusted RORs and adjusted RORs were statistically significant. Nevertheless, the possibility of alternative explanations, including unmeasured confounding and reporting artifacts, remains. Our regimen‑stratified approach and large sample size enhance the breadth of the analysis, but they do not compensate for the absence of randomized allocation or detailed clinical data. Consequently, the present results should be viewed as complementary to, rather than a replacement for, evidence from well‑designed prospective cohort studies and randomized controlled trials.

## Conclusions

Cholecalciferol supplementation demonstrated favorable reporting trends regarding transplant outcomes within standard maintenance regimens, while higher reporting signal frequencies were observed in MAB-containing protocols. These findings reflect associations in disproportional reporting rather than proven effects on clinical risk and should be interpreted cautiously, considering the inherent limitations of spontaneous reporting databases. Clinicians may take these observations into account when evaluating supplementation strategies in high-risk RTRs undergoing induction or rejection therapy. As these are hypothesis‑generating findings derived from an observational pharmacovigilance database, prospective and adequately powered randomized controlled trials are warranted to further elucidate the potential role of cholecalciferol across diverse immunosuppressive contexts.

## Appendices

Appendix A

**Table 6:** Data handling for patient background variables.

Data handling for patient background variables
Sex: We treated {gndr_cod} and {sex} as equivalent variables, and combined them into a single {sex} variable. Entries other than “M” or “F”, as well as missing values, were categorized as “unknown”. Age: The {age} variable was reported in various units indicated by {age_cod} (age unit code), including years, decades, months, weeks, or days. To standardize age data to years, we applied the following conversion rules: (1) Decades: multiplied the reported value by 10 and added 5 to approximate the midpoint of the decade. (2) Months: divided by 12 and rounded down to the nearest whole number. (3) Weeks: divided by (365.25/7) and rounded down to the nearest whole number. (4) Days: divided by 365.25 and rounded down to the nearest whole number. Weight: The {wt} (weight) variable was primarily recorded in kilograms, with units indicated by {wt_cod} (weight unit code). Weights reported in pounds were converted to kilograms by multiplying the value by 0.454 (kg per pound). Reporter country: Reports with {reporter_country} coded as “COUNTRY NOT SPECIFIED” were classified as “unknown”. In descriptive summaries (Table 1), {reporter_country} was categorized into six continents (North America, Latin America, Asia, Europe, Oceania, and Africa), with “unknown” as a separate category. For binomial logistic regression analyses (Tables 3 and 4), {reporter_country} was further collapsed into three regions (North America, Europe, and other continents) as described in the main text.

Appendix B

**Table 7:** Generic and brand names of immunosuppressants and cholecalciferol.

Generic name	Brand name
Calcineurin inhibitors (CNIs)	
Tacrolimus	Adoport, Advagraf, Astagraf, Envarsus, Graceptor, Prograf, Tacsant
Cyclosporine	Ciclosporin, Ciclosporine, Cyclosporin, Equoral, Gengraf, Neoral, Sandimmune
Steroids (STRs)	
Prednisone, Prednisolone, Methylprednisolone	Aprednislon, Cortancyl, Decortin, Medrol, Prednisolon, Prednison, Prednisona, Predonine, Solupred, Urbason
Antimetabolites (ANTs)	
Mycophenolate mofetil, Mycophenolic acid	Cellcept, Myfortic
Azathioprine	Azanin, Azasan, Azathioprin, Imuran, Imurek, Imurel, Zytrim
Mizoribine	Bredinin
Mechanistic target of rapamycin inhibitors (mTOR)	
Everolimus	Afinitor, Certican, Rad001, Sdz-rad, Zortre
Monoclonal antibodies (MABs)	
Rituximab	Mabthera, Rituxan
Basiliximab	Simulect
Cholecalciferol	Colecalciferol, Vitamin D, Vitamin D3

Appendix C

Identification of potential duplicate reports

Because FAERS accepts reports from multiple reporter types, we attempted to identify potential duplicate reports referring to the same individual. We considered reports to be potential duplicates when all of the following FAERS variables were identical: {drugname}, {sex}, {age}, {age_cod}, {wt}, {wt_cod}, {reporter_country}, {start_dt} (date therapy was started (or re-started) for this drug), {end_dt} (date therapy was stopped for this drug), {event_dt} (date the adverse event occurred or began), and {pt}. When such potential duplicates were detected, only a single record was retained per patient for the analyses.

Appendix D

**Table 8:** Categorization of preferred terms.

Adverse event category	Preferred terms
Transplant outcome events	Complications of transplanted kidney, Graft dysfunction, Graft loss, Transplant rejection
Infections	Adenovirus infection, Bacterial infection, BK virus infection, Candida infection, Cellulitis, COVID-19, COVID-19 pneumonia, Cytomegalovirus infection, Cytomegalovirus viraemia, Enterococcal infection, Epstein-Barr virus infection, Escherichia infection, Escherichia urinary tract infection, Fungal infection, Herpes zoster, Infection, Influenza, Kidney infection, Klebsiella infection, Lung infection, Nocardiosis, Pneumocystis jirovecii pneumonia, Polyomavirus-associated nephropathy, Pseudomonas infection, Sepsis, Septic shock, Staphylococcal infection, Urinary tract infection, Urosepsis, Viral infection
Cancer	Basal cell carcinoma, Kaposi's sarcoma, Post transplant lymphoproliferative disorder, Skin cancer
Renal disorders	Acute kidney injury, Anuria, Blood creatinine increased, Blood urea increased, Chronic allograft nephropathy, Delayed graft function, Dialysis, Focal segmental glomerulosclerosis, Haemodialysis, Hydronephrosis, Kidney fibrosis, Nephropathy toxic, Proteinuria, Pyelonephritis, Renal disorder, Renal failure, Renal failure acute, Renal impairment, Renal tubular atrophy, Renal tubular necrosis, Tubulointerstitial nephritis, Urine output decreased
Musculoskeletal and mobility disorders	Back pain, Fall, Gait disturbance, Muscular weakness, Myalgia, Pain, Pain in extremity
General disorders	Asthenia, Chills, Erythema, Fatigue, General physical health deterioration, Insomnia, Malaise, Oedema peripheral, Pyrexia, Rash, Swelling
Gastrointestinal disorders	Abdominal distension, Abdominal pain, Abdominal pain upper, Ascites, Constipation, Decreased appetite, Diarrhoea, Dysphagia, Gastrointestinal disorder, Gastrointestinal haemorrhage, Nausea, Pruritus, Vomiting, Weight decreased, Weight increased
Cardiovascular disorders	Atrial fibrillation, Blood pressure increased, Cardiac arrest, Cardiac disorder, Cardiac failure, Cardiac failure congestive, Cerebrovascular accident, Deep vein thrombosis, Hypertension, Hypotension, Myocardial infarction, Pulmonary embolism, Tachycardia, Thrombosis
Hematologic disorders	Agranulocytosis, Anaemia, C-reactive protein increased, Haemoglobin decreased, Leukocytosis, Leukopenia, Lymphocyte count decreased, Lymphopenia, Neutropenia, Pancytopenia, Platelet count decreased, Thrombocytopenia, White blood cell count decreased, White blood cell count increased
Nervous system disorders	Coma, Confusional state, Dizziness, Headache, Neurotoxicity, Seizure, Tremor
Respiratory disorders	Acute respiratory distress syndrome, Cough, Dyspnoea, Lung disorder, Pleural effusion, Pneumonia, Productive cough, Pulmonary oedema, Respiratory failure
